# *Calidifontibacillus erzuremensis* , *Pantoea agglomerans* , and *Pseudomonas glycinae* identified as antibiotic-producers from freshwater

**DOI:** 10.17912/micropub.biology.001362

**Published:** 2024-11-25

**Authors:** Caymen Hoffman, Kristina Blanke

**Affiliations:** 1 Beloit College, Beloit, Wisconsin, United States; 2 Pakula Biomedical Fellowship

## Abstract

Three water isolates were previously identified as promising antibiotic producers from freshwater sources in Wisconsin, United States. Each isolate produced effective antibiotics against three or more bacterial relatives of antibiotic resistant pathogens. The isolates were identified as *Calidifontibacillus erzuremensis* , *Pantoea agglomerans* , and *Pseudomonas glycinae* through 16S rRNA sequencing and further characterized with biochemical tests to verify the genus and species of each isolate.

**Figure 1. Characteristics of three freshwater isolates with antibiotic activity against three or four tester bacteria related to antibiotic resistant pathogens:**
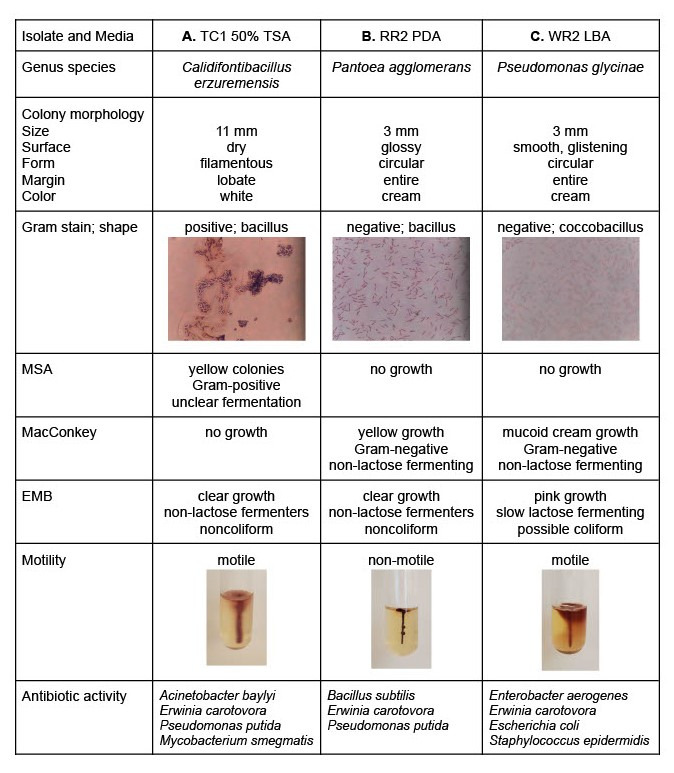
Each isolate is labeled based on their sampling location and the isolate number: TC = Turtle Creek, RR = Rock River, WR = Wisconsin River. Culture media included 50% tryptic soy agar (50% TSA), potato dextrose agar (PDA), and lysogeny broth agar (LBA). The selective/differential media was mannitol salt agar (MSA), MacConkey agar, and eosin-methylene blue (EMB) agar. **Column A.** Isolate TC1 was cultured on 50% TSA and has characteristics aligning with *Calidifontibacillus erzuremensis* . TC1 produced antibiotics that inhibited the growth of four Gram-negative or acid fast tester bacteria. **Column B.** Isolate RR2 was cultured on PDA and has characteristics aligning with *Pantoea agglomerans* . RR2 produced antibiotics that inhibited the growth of three Gram-positive or Gram-negative tester bacteria. **Column C.** Isolate WR2 was cultured on LBA and has characteristics aligning with *Pseudomonas glycinae* . WR2 produced antibiotics that inhibited the growth of four Gram-positive or Gram-negative tester bacteria.

## Description

Freshwater from creeks and rivers in Wisconsin contained bacteria that produced antibiotics against Gram-negative, Gram-positive, and acid fast relatives of antibiotic resistant pathogens (Hoffman et al., 2024) . Several water isolates inhibited the growth of three or four tester bacteria through a spread/patch screen and warranted further investigation. This study identified three isolates with 16S rRNA sequencing and verified the results with their biochemical characteristics. The lethal outcomes for each isolate against multiple tester bacteria emphasize the need to investigate novel environments to discover new natural classes of antibiotics during the global healthcare crisis of rising antibiotic resistant pathogens.

Isolate TC1 was cultured on 50% TSA and identified as *Calidifontibacillus erzuremensis* ( Figure 1 .A). TC1 was grown in aerobic conditions, stained Gram-positive with a bacillus shape, and was motile; which aligns with the known characteristics of *C. erzuremensis* (Adiguzel et al., 2020) . TC1 growth on 50% TSA led to white colonies with a filamentous form and lobate margin, further correlating with the expected morphology of *C. erzuremensis* on TSA (Adiguzel et al., 2020) . TC1 grew as yellow colonies on MSA, indicating this isolate is Gram-positive and can tolerate higher levels of salt in media, as is the case for *C. erzuremensis* (Adiguzel et al., 2020) . TC1 also grew as clear colonies on EMB, which demonstrates a lack of lactose and sucrose fermentation and follows the expected characteristics of *C. erzuremensis* (Adiguzel et al., 2020) . Additional data gathered for TC1 showed antibiotic activity against four tester bacteria: Gram-negative *A. baylyi* , *E. carotovora* , *P. putida* ; acid fast *M. smegmatis* . *C. erzuremensis* is a novel species that has not been thoroughly studied and the antibiotic activity results are encouraging that the secondary metabolites from this species have pharmaceutical potential.

Isolate RR2 was cultured on PDA and identified as *Pantoea agglomerans* ( Figure 1 .B). RR2 grew in aerobic conditions, stained Gram-negative with a bacillus shape, was isolated from a water source, and appeared glossy with a circular form and entire margin; which aligns with expected characteristics of *P. agglomerans* (Gavini et al., 1989) . RR2 was confirmed as a Gram-negative species from yellow colony growth on MacConkey agar and clear growth on EMB. The MacConkey and EMB agar results indicate RR2 is a non-lactose fermenter. *P. agglomerans* is known to be motile (Gavini et al., 1989) ; however, isolate RR2 was non-motile. Additional data gathered for RR2 showed antibiotic activity against three tester bacteria: Gram-positive *B. subtilis* ; Gram-negative *E. carotovora* , *P. putida* . Previous studies found one strain of *P. agglomerans* produced two antibiotics (pantocin A and B) that inhibited growth of *E. carotovora* (Wright et al., 2001) . The antibiotics produced by RR2 may include pantocin A and B, which could be effective against *B. subtilis* and *P. putida* . However, it is unclear which secondary metabolites RR2 produces as antibiotics since antiSMASH 7.0 identify four main secondary metabolites for *P. agglomerans* (ASM1904838v1) with 84-100% similarity that included metal sequestering (frederiksenibactin and desferrioxamine) or pigmentation (aryl polyenes and carotenoid) molecules (Blin et al., 2023) , though there were no gene clusters for pantocin.

Isolate WR2 was cultured on LBA and identified as *Pseudomonas glycinae* ( Figure 1 .C). WR2 grew in aerobic conditions, stained Gram-negative with a coccobacillus shape, was motile, and catalase positive; which follows the expected characteristics of *P. glycinae* (Jia et al., 2020) . The colony morphology of WR2 also aligned with *P. glycinae* characteristics of being 3 mm in diameter with a circular shape, entire margin, and cream color on LBA (Jia et al., 2020) . WR2 was confirmed as a Gram-negative species from mucoid cream growth on MacConkey agar and the results indicated that WR2 was not fermenting lactose; however, the EMB agar results showed pink growth and indicated that WR2 was a slow lactose fermenting bacteria and a possible coliform. In this study, WR2 showed antibiotic activity against four tester bacteria: Gram-positive *S. epidermidis* ; Gram-negative *E. aerogenes* , *E. carotovora* , *E. coli* . *P. glycinae* is another novel species that needs a thorough investigation and the additional data for antibiotic activity indicate the secondary metabolites for this species also have pharmaceutical potential.

In conclusion, *Calidifontibacillus erzuremensis* , *Pantoea agglomerans* , and *Pseudomonas glycinae* were discovered in Wisconsin freshwater and produced antibiotics that inhibited the growth of Gram-positive, Gram-negative, and acid fast bacteria.

## Methods

Sample Selection

Water samples were collected from four water systems in Wisconsin, United States (Hoffman et al., 2024) , and screened against tester bacteria provided by the Tiny Earth organization (Hoffman & Blanke, 2024; Hernandez et al., 2018) . Colonies chosen for screening were categorized by size, surface, form, margin, and color. Additional protocol information regarding the collection, screening, and identification of antibiotic-producing bacteria from the environment is described in the Hoffman and Blanke (2024) article. Three isolates were selected for further analysis since they produced antibiotics against 3-4 tester bacteria across two of the three bacterial categories: Gram positive, Gram negative, acid fast. Each sample was from a different water system (Turtle Creek, Rock River, Wisconsin River) and cultured on various media (50% TSA, PDA, LBA, respectively) as described by Hoffman et al. (2024).

Genus species Identification

Each isolate was prepared for 16S rRNA sequencing (Hernandez et al., 2018) performed by Eurofins Genomics (Louisville, KY). Sequencing text was run through BLASTn (courtesy of the National Library of Medicine) and the genus species for each isolate was chosen based on the highest percent identify and alignment (Table 1).

**Table 1.** Isolate 16S rRNA sequences identified the genus and species. The corresponding percent identity and number of aligned base pairs (bp) are listed as compiled by BLASTn.

**Table:** 

Isolate	Genus species	Reference	Percent Identity	Alignment
TC1	*Calidifontibacillus erzuremensis*	NR_180225.1	99.52%	837 bp
RR2	*Pantoea agglomerans*	NR_041978.1	96.82%	1125 bp
WR2	*Pseudomonas glycinae*	NR_179889.1	100.00%	924 bp

16S rRNA sequence

TC1 sequence:

AACACGTGGGTAACCTGCCTGTAAGACTGGGATAACTCCGGGAAACCGGGGCTAATACCGGATGGTTGTTTGAACCGCATGGTTCAGACATAAAAGGTGGCTTCGGCTACCACTTACAGATGGACCCGCGGCGCATTAGCTAGTTGGTGAGGTAACGGCTCACCAAGGCGACGATGCGTAGCCGACCTGAGAGGGTGATCGGCCACACTGGGACTGAGACACGGCCCAGACTCCTACGGGAGGCAGCAGTAGGGAATCTTCCGCAATGGACGAAAGTCTGACGGAGCAACGCCGCGTGAGTGATGAAGGTTTTCGGATCGTAAAGCTCTGTTGTTAGGGAAGAACAAGTGCCGTTCAAATAGGGCGGCACCTTGACGGTACCTAACCAGAAAGCCACGGCTAACTACGTGCCAGCAGCCGCGGTAATACGTAGGTGGCAAGCGTTGTCCGGAATTATTGGGCGTAAAGGGCTCGCAGGCGGTTTCTTAAGTCTGATGTGAAAGCCCCCGGCTCAACCGGGGAGGGTCATTGGAAACTGGGGAACTTGAGTGCAGAAGAGGAGAGTGGAATTCCACGTGTAGCGGTGAAATGCGTAGAGATGTGGAGGAACACCAGTGGCGAAGGCGACTCTCTGGTCTGTAACTGACGCTGAGGAGCGAAAGCGTGGGGAGCGAACAGGATTAGATACCCTGGTAGTCCACGCCGTAAACGATGAGTGCTAAGTGTTAGGGGGTTTCCGCCCCTTAGTGCTGCAGCTAACGCATTAAGCACTCCGCCTGGCGAGTACGGTCGCAAGACTGAAACTCAAAGGAACGGACGGAGGCCCGCACAAGCGGTGGAG

RR2 sequence:

CTAACACATGCAGAGTCGAACGGTAGCACAGAGAGCTTGCTCTTGGGTGACGAGTGGCGGACGGGTGAGTAATGTCTGGGGATCTGCCTGACAGAGGGGGATAACTACTGGAAACGGTAGCTAATACCGCATAACCTCGCAAGAGCAAAGAGGGGGACCTTCGGGCCTCTCGCTGTCAGATGAACCCAGATGGGATTAGCTAGTAGGTGGGGTAATGGCTCACCTAGGCGACGATCCCTAGCTGGTCTGAGAGGATGACCAGCCACACTGGAACTGAGACACGGTCCAGACTCCTACGGGAGGCAGCAGTGGGGAATATTGCACAATGGGCGCAAGCCTGATGCAGCCATGCCGCGTGTATGAAGAAGGCCTTCGGGTTGTAAAGTACTTTCAGCGGGGAGGAAGGTGATGAGGTTAATAACCTCGTCAATTGACGTTACCCGCAGAAGAAGCACCGGCTAACTCCGTGCCAGCAGCCGCGGTAATACGGAGGGTGCAAGCGTTAATCGGAATTACTGGGCGTAAAGCGCACGCAGGCGGTCTGTCAAGTCAGATGTGAAATCCCCGGGCTTAACCTGGGAACTGCATTTGAAACTGGCAGGCTAGAGTCTTGTAGAGGGGGGTAGAATTCCAGGTGTAGCGGTGAAATGCGTAGAGATCTGGAGGAATACCGGTGGCGAAGGCGGCCCCCTGGACAAAGACTGACGCTCAGGTGCGAAAGCGTGGGGAGCAAACAGGATTAGATACCCTGGTAGTCCACGCCGTAAACGATGTCGACTTGGAGGTTGTTCCCTTGAGGAGTGGCTTCCGGAGCTAACGCGTTAAGTCGACCGCCTGGGGAGTACGGCCGCAAGGTTAAAACTCAAATGAATTGACGGGGGCCCGCACAAGCGGTGGAGCATGTGGTTTAATTCGATGCAACGCGAAGAACCTTACCTACTCTTGACATCCACAGAATTTGGCAGAGATGCCTTGGTGCCTTCGGGAACTCTGAGACAGGTGCTGCATGGCTGTCGTCAGCTCGTGTTGTGAAATGTTGGGTTAAGTCCCGCAACGAGCGCAACCCTTATCTTTGTTGCCACACGTAATGGTGGGAACTCAAAGGAGACTGCCGGTGATAACCGGAGGAAGGGGGGGATGACGTCAGTNATCATG

WR2 sequence:

CCTAGGAATCTGCCTGGTAGTGGGGGACAACGTTTCGAAAGGAACGCTAATACCGCATACGTCCTACGGGAGAAAGCAGGGGACCTTCGGGCCTTGCGCTATCAGATGAGCCTAGGTCGGATTAGCTAGTTGGTGAGGTAATGGCTCACCAAGGCGACGATCCGTAACTGGTCTGAGAGGATGATCAGTCACACTGGAACTGAGACACGGTCCAGACTCCTACGGGAGGCAGCAGTGGGGAATATTGGACAATGGGCGAAAGCCTGATCCAGCCATGCCGCGTGTGTGAAGAAGGTCTTCGGATTGTAAAGCACTTTAAGTTGGGAGGAAGGGTTGTAGATTAATACTCTGCAATTTTGACGTTACCGACAGAATAAGCACCGGCTAACTCTGTGCCAGCAGCCGCGGTAATACAGAGGGTGCAAGCGTTAATCGGAATTACTGGGCGTAAAGCGCGCGTAGGTGGTTCGTTAAGTTGGATGTGAAATCCCCGGGCTCAACCTGGGAACTGCATCCAAAACTGGCGAGCTAGAGTATGGTAGAGGGTGGTGGAATTTCCTGTGTAGCGGTGAAATGCGTAGATATAGGAAGGAACACCAGTGGCGAAGGCGACCACCTGGACTGATACTGACACTGAGGTGCGAAAGCGTGGGGAGCAAACAGGATTAGATACCCTGGTAGTCCACGCCGTAAACGATGTCAACTAGCCGTTGGGAGCCTTGAGCTCTTAGTGGCGCAGCTAACGCATTAAGTTGACCGCCTGGGGAGTACGGCCGCAAGGTTAAAACTCAAATGAATTGACGGGGGCCCGCACAAGCGGTGGAGCATGTGGTTTAATTCGAAGCAACGCGAAGAACCTTACCAGGCCTTGACATCCAATGAACTTTCCAGAGATGGATTGGTGCCTTCGGGAACATTGAGACA

Gram Staining and Selective/Differential Media

All isolates were categorized by their shape and cell wall structure through Gram staining. Additional selective/differential media tests were performed to confirm Gram staining results and identify isolate fermentation capabilities. MacConkey and EMB agars confirmed Gram-negative isolates, and MSA confirmed select Gram-positive isolates. MacConkey agar identified lactose fermentation, EMB agar identified lactose and sucrose fermentation, and MSA identified mannitol fermentation. EMB agar also indicated coliform status for any isolates with growth. Each isolate was cultured on either 50% TSA or LBA as a control for the selective/differential media tests. Media were incubated at 30°C with light exposure for 24-48 hours after inoculation.

Motility

Isolates were inoculated in general media with 2,3,5-triphenyl tetrazolium to visualize bacterial movement and incubated at 25°C for seven days. Isolates with spreading growth were motile, while non-motile isolates grew along the insertion line. Results were either motile or non-motile.

## References

[R1] Adiguzel Ahmet, Ay Hilal, Baltaci Mustafa Ozkan, Akbulut Sumeyya, Albayrak Seyda, Omeroglu Mehmet Akif (2020). Genome-based classification of Calidifontibacillus erzurumensis gen. nov., sp. nov., isolated from a hot spring in Turkey, with reclassification of Bacillus azotoformans as Calidifontibacillus azotoformans comb. nov. and Bacillus oryziterrae as Calidifontibacillus oryziterrae comb. nov.. International Journal of Systematic and Evolutionary Microbiology.

[R2] Blin Kai, Shaw Simon, Augustijn Hannah E, Reitz Zachary L, Biermann Friederike, Alanjary Mohammad, Fetter Artem, Terlouw Barbara R, Metcalf William W, Helfrich Eric J N, van Wezel Gilles P, Medema Marnix H, Weber Tilmann (2023). antiSMASH 7.0: new and improved predictions for detection, regulation, chemical structures&nbsp;and visualisation. Nucleic Acids Research.

[R3] GAVINI F., MERGAERT J., BEJI A., MIELCAREK C., IZARD D., KERSTERS K., DE LEY J. (1989). Transfer of Enterobacter agglomerans (Beijerinck 1888) Ewing and Fife 1972 to Pantoea gen. nov. as Pantoea agglomerans comb. nov. and Description of Pantoea dispersa sp. nov.. International Journal of Systematic Bacteriology.

[R4] Hernandez S, Tsang T, Bascom-Slack C, Broderick N, and Handelsman J. 2018. Tiny Earth: A Research Guide to Student sourcing Antibiotic Discovery. Livonia (MI): XanEdu Publishing Inc. ISBN: 978-1-71147-072-6

[R5] Hoffman Caymen, Blanke Kristina (2024). Five tree species contained antibiotic-producing bacteria within their bark.

[R6] Hoffman Caymen, Caracoza Edgar, Kyaw Kevin, Blanke Kristina (2024). Seventeen antibiotic-producing bacteria isolates found across four freshwater environments.

[R7] Jia Jiayuan, Wang Xiaoqiang, Deng Peng, Ma Lin, Baird Sonya M., Li Xiangdong, Lu Shi‐En (2020). *Pseudomonas glycinae*
sp. nov. isolated from the soybean rhizosphere. MicrobiologyOpen.

[R8] Wright Sandra A. I., Zumoff Cathy H., Schneider Lois, Beer Steven V. (2001). *Pantoea agglomerans*
Strain EH318 Produces Two Antibiotics That Inhibit
*Erwinia amylovora*
In Vitro. Applied and Environmental Microbiology.

